# Life Expectancy in Asian Muslim-Majority Countries: A Comparative Perspective

**DOI:** 10.34172/hpp.44337

**Published:** 2026-06-06

**Authors:** Mohammad Shafi Mohammadi

**Affiliations:** Department of Economics, School of Economics, Central University of Kerala, Kasaragod, Kerala, India

**Keywords:** Life expectancy, Asian Muslim majority countries, Mortality rate, Fertility rate, Infant death rate

## Abstract

This study examines life expectancy in Asian Muslim-majority countries from a comparative perspective, with a particular focus on the role of socioeconomic and health-related determinants. Using secondary data from the World Bank and other international databases across countries which are categorized by income levels. The findings reveal substantial disparities among countries, where high-income Gulf nations such as Qatar, the United Arab Emirates, and Kuwait exhibit higher life expectancy, lower mortality, and improved child health outcomes, primarily due to advanced healthcare systems and economic prosperity. In contrast, low-income and conflict-affected countries such as Afghanistan, Yemen, and Pakistan experience lower life expectancy, higher mortality, and elevated fertility rates, driven by poverty, political instability, and limited access to healthcare services. Middle-income countries demonstrate transitional patterns with gradual improvements in health indicators. Overall, the study highlights a strong association between economic status and life expectancy, emphasizing the need for targeted health policies, improved healthcare access, and socioeconomic development to reduce disparities across the region. These findings provide important insights for policymakers aiming to enhance population health and promote equitable development in Asian Muslim-majority countries.

 Over the past century, life expectancy has steadily increased, or the number of years that a person may expect to live.^1^ Life expectancy is critical for assessing population health and well-being, developing public health efforts, and establishing development targets. Life expectancy varies between countries with differing economic backgrounds. Thus, life expectancy varies by social class, with wealthy people living longer and better lives.^2^ Furthermore, these shifts are occurring in Asian Muslim-majority, prosperous countries, which are projected to have a longer life expectancy than poor countries. To better understand these inequalities, we must examine how economic power, as measured by per capita GDP, influences life expectancy in Muslim-majority countries.

 Higher per-capita GDP has a significant positive impact on life expectancy.^3^ The wealthiest Muslim-majority countries in Asia, according to per-capita GDP, include Qatar (USD 87,566.5), the United Arab Emirates (USD 53,708), Brunei (USD 37,152.5), Kuwait (USD 41,079.5), Saudi Arabia (USD 30,447.9), Bahrain (USD 30,146.6), and Oman (USD 25,056.5).^4^ These nations, mostly in the Gulf region, have high-income economies, small populations, and advanced infrastructure and have higher life expectancy.^5^ In contrast, the poorest countries include Afghanistan (USD 355.8), Syria (USD 420.6), Yemen (USD 650.3), Tajikistan (USD 1,054.2), Pakistan (USD 1,588.9), and Kyrgyzstan (USD 1,655.1);^4^ have the highest population,^5^ and the lowest life expectancy among the Muslim-majority countries. These economic contrasts are directly reflected in the life expectancy levels observed across the region, highlighting how national wealth translates into population health outcomes.

 The world average life expectancy at birth is 71 years.^6^ The highest life expectancies among the Asian Muslim-majority were found in high-income Gulf states like Qatar (81 years), the United Arab Emirates (79 years), Kuwait (80 years), Bahrain (79 years), and Saudi Arabia (77 years), where advanced healthcare systems and higher incomes contribute to longer lifespans.^7^ Middle-income countries like Malaysia (76 years), Turkey (78 years), and Iran (74 years) also show strong performance, supported by improving health infrastructure and socioeconomic growth.^7^ In contrast, life expectancies are lower in low-income countries like Afghanistan (62 years), Yemen (63 years), and Syria (72 years), because of persistent conflict, poverty, and a lack of access to quality healthcare.^7^ Additionally, Indicators of mortality offer more information about the health of the community.

 High-income nations with advanced medical systems and healthier lifestyles and less mortality, like Saudi Arabia (Male: 95, Female: 76), Kuwait (Male: 55, Female: 29), Bahrain (Male: 59, Female: 50), the United Arab Emirates (Male: 68, Female: 45), and Qatar (Male: 42, Female: 34), had the lowest adult mortality rates (out of 1000 individuals).^8^ On the contrary, low-income and conflict-affected countries such as Afghanistan (Male: 329, Female: 203), Yemen (Male: 296, Female: 171), and Syria (Male: 199, Female: 90) out of 1000 individuals recorded the highest mortality rates, largely due to war, poor healthcare infrastructure, and economic instability.^6^ Moderate rates are seen in middle-income nations like Malaysia, Iran, and Turkey, which reflect steady advancements in life expectancy and healthcare.^8^ Moreover, examining child mortality, one of the most sensitive measures of a country’s health and development, reveals the same pattern.

 The lowest rates of child mortality were observed in high-income Gulf countries such as the United Arab Emirates (Male: 4, Female: 3), Qatar (Male: 4, Female: 4), Bahrain (Male: 5, Female: 5), Kuwait (Male: 8, Female: 6), and Saudi Arabia (Male: 5, Female: 5) out of 1000 children, reflecting advanced healthcare systems, strong maternal services, and high living standards.^9^ Middle-income countries like Turkey (Male: 8, Female: 7), Iran (Male: 10, Female: 9), and Malaysia (Male: 7, Female: 6) also recorded low mortality, showing steady progress in child health and healthcare access.^9^ Afghanistan (Male: 48, Female: 41), Pakistan (Male: 55, Female: 46), and Yemen (Male: 36, Female: 29) are examples of low-income and conflict-affected nations that continue to have high infant mortality rates as a result of inadequate medical infrastructure, malnutrition, and instability.^9^ Beyond mortality statistics, differences in fertility and death rates also highlight the health outcomes of Asian Muslim-majority countries.

 Death and fertility rates are vital indicators that shape population growth and structure while reflecting a country’s health and living standards. Among Asian high income Muslim-majority nations such as Qatar (death: 1; fertility: 1), the United Arab Emirates (death: 1; fertility: 1), Kuwait (death: 2; fertility: 2), Bahrain (death: 2; fertility: 1), and Saudi Arabia (death: 2; fertility: 2) recorded the lowest death and fertility rates due to advanced healthcare systems and improved living standards.^10^ Middle-income countries like Iran (death: 6; fertility: 1), Turkey (death: 5; fertility: 1), and Malaysia (death: 5; fertility: 1) exhibited moderate levels, reflecting progress in healthcare and education that influence family planning.^10^ Oppositely, low-income and conflict-affected nations such as Afghanistan (death: 6; fertility: 4), Yemen (death: 6; fertility: 3), and Pakistan (death: 7; fertility: 3) showed higher rates, indicating persistent poverty, limited health facilities, and traditional reproductive norms.^10^ Taken together, these indicators demonstrate how economic status, healthcare infrastructure, and social stability collectively shape life expectancy outcomes across the Asian Muslim-majority countries.

 In general, life expectancy and mortality patterns across Asian Muslim-majority countries clearly reflect the strong link between economic development, healthcare access, and overall well-being. High-income Gulf nations, such as Qatar, the United Arab Emirates, Kuwait, Bahrain, and Saudi Arabia, demonstrate the highest life expectancy, the lowest mortality rates, and minimal infant mortality, driven by robust healthcare systems, economic prosperity, and improved living conditions. Middle-income countries, such as Iran, Turkey, and Malaysia, exhibit steady improvement, marking a transition toward lower fertility and mortality rates as their healthcare infrastructure and education systems advance. Conversely, low-income and conflict-affected nations such as Afghanistan, Yemen, and Pakistan continue to experience poor health outcomes, high mortality, and elevated fertility rates, primarily due to inadequate healthcare access, poverty, and instability. Overall, the data illustrate a pronounced socioeconomic divide, where wealthier Muslim-majority nations enjoy longer, healthier lives, while poorer countries remain burdened by preventable deaths and limited medical resources, emphasizing the urgent need for equitable investment in health and development systems across the poor regions.

## Future Discourse

 In the future, the discussion of life expectancy in Muslim-majority Asian countries should concentrate on closing the growing gap between high- and low-income countries. Future research must examine how specific legislative changes, fair resource allocation, and medical technology developments can lengthen life expectancy in states with weaker economies. International cooperation will be essential to reducing disparities, especially in the areas of infrastructure development, education, and medical research. Furthermore, creating more inclusive strategies can be aided by knowing how gender equity, governance quality, and cultural factors affect health outcomes. Prioritizing health equity will be crucial as nations work toward the Sustainable Development Goals in order to guarantee that increases in life expectancy are distributed among all social and economic groups.

## Competing Interests

 The author declares that they have no competing interests.

## Ethical Approval

 Not applicable.
